# Establishment of a Research Unit in Colac, a Medium Rural Town: An Update on Progress and Guidance for Rural Health Service Research Strategy Development

**DOI:** 10.1111/ajr.70005

**Published:** 2025-02-07

**Authors:** Laura Alston, Michael Field, Alison Buccheri, Fiona Brew, Anna George, Nikita Wheaton, Stella Harrington, Warren Payne, Drew Aras, Alice Bennett, Hannah Beks, Kevin Mc Namara, Vincent L. Versace

**Affiliations:** ^1^ Research Unit Colac Area Health Colac Victoria Australia; ^2^ Deakin Rural Health, School of Medicine Deakin University Warrnambool Victoria Australia; ^3^ Western Alliance Academic Health Science Centre Warrnambool Victoria Australia

**Keywords:** place‐based research, rural health, rural health services

## Abstract

**Background:**

Rural health services have not had the same opporunities for research as metropolitan health services. Despite increasing awareness of the importance of placed‐based research led by rural health services, there are few examples in the literature on how this can be done.

**Aims:**

In this AJRH Practice Insight we aim to provide an update on the establishment and progress of the Colac Area Health (CAH) research unit. This is a health service led research unit that services rural areas classified as MM4‐5 by the Modified Monash Model.

**Methods:**

Practice insight.

**Discussion:**

This experience may assist other small or medium‐sized rural health services to undertake the same strategic goal of building locally relevant and place‐based research, along with providing hope for those setting out to integrate research into rural health service organisational structures, while minimising the burden on existing resources.

**Conclusion:**

This Practice Insight demonstrates that rural health services can integrate research units into their organisational structures, with minimal burden on resources alongside support from partners who understand the value of rural health research. Lessons learned serve as a valuable example for other rural health services who are seeking to drive their own research programs and support their staff to access research career opportunities.

**Summary**

- What this paper adds
  ○ Metropolitan hospital staff members have enjoyed the opportunity of having embedded research departments for decades, and unfortunately, the same cannot be said for rural health services.
  ○ This paper provides an update on the Colac Area Health Research Unit and outlines a practical example of how small or medium‐sized rural health services can undertake the strategic goal of building locally relevant and place‐based research.
- What is already known on this subject
  ○ Place‐based research has been increasingly acknowledged as important when undertaking rural health research, to ensure the involvement of the local community. With an increasing attention on the need for place‐based research, there is focus on the ability and capacity of rural health services to lead their own research programs.
  ○ Practical examples of how this can be done are scarce in the scholarly literature, meaning that there is minimal guidance for how rural health services can embed research into ‘usual business’ for their organisational structures.

Metropolitan‐based hospitals typically have substantial resources, networks and infrastructure to support research and enable research career opportunities for metropolitan based clinicians [1]. Staff at rural health services have not enjoyed the same research opportunities, translating to lower research and career progression opportunities for the rurally based health workforce [2, 3]. In recent years, Australian rural health services have clearly demonstrated that they are critical settings for research exploration, delivery and evidence translation [3, 4, 5, 6]. This is due to smaller organisational structures and demonstrated ability to rapidly implement changes arising from research, supporting a more immediate influence and increased impact from research efforts [4, 5, 6, 7, 8]. Health benefits resulting from the impact of research are much needed in rural areas [4].

Growing research capacity in rural health services not only has direct benefits for career opportunities for the local rural workforce but also the delivery of evidence‐based care and fostering cultures of innovation [4]. Building research culture and capability in rural health services needs to be context specific [9] to assist with addressing equity issues, such as ensuring rural communities are appropriately represented in clinical trials [10], along with building evidence that is appropriate to the rural context.

Despite increasing awareness of the role that rural health services can play in understanding the best ways to address rural health gaps through driving their own research programs, there are few examples in the literature on how this can be practically done. In this *AJRH Practice Insight*, we provide an update on the establishment and progress of the Colac Area Health (CAH) research unit, following our practice insight paper published in AJRH in 2022 [7]. The aim of disseminating this experience is to assist other small or medium‐sized rural health services to undertake the same strategic goal of building locally relevant and place‐based research, along with providing hope for those setting out to integrate research into rural health service organisational structures, while minimising the burden on existing resources.

## 1 Update and Progress

Colac Area Health services communities defined as both ‘medium rural towns’ (Category 4) and ‘small rural towns’ (Category 5) by the Modified Monash Model (MMM) [11]. In comparison with other health services of this size, CAH has made significant progress in establishing a research unit within its organisational and governance structures [12]. As the publication of our practice insight in 2022, the rural health service research unit has made further progress, building on funding support from Deakin Rural Health (the local University Department of Rural Health, UDRH), the Western Alliance Academic Health Science Centre and partnerships with local communities, clinicians and established career researchers. Key to continuing the development of the CAH research unit has been committed health service leadership, collaboration with external partners, inclusion in grant proposals by career researchers, the development of an organisational research strategy and competitive research funding. Although we acknowledge that securing funding for research positions will be a challenge for most rural health services, long‐term partnerships have been key to success in this example.

Through collaboration with career researchers leading large competitive grants, the research unit has secured further funding to support the delivery of research on the ground that is relevant to the rural context. This has seen our research unit go from a team of two individuals, to up to a team of eight staff members in 2023, and the unit has external funding for 2.4 full time equivalent researchers in the 2024–2025 financial year. In 2022, we reported that the health service was a leader or partnering site in almost 30 projects, and now this has expanded to over 50 research projects spanning multiple topics that are highly relevant to the local health service and community. Anecdotally, internal success stories from small research projects have assisted the research unit with sustaining a culture shift towards research, building local capacity and skills, and led to improvements in local policy and practice. Preliminary evaluation data also show positive impacts on workforce satisfaction and career opportunities for staff who have engaged with the research unit.

## 2 Organisational Research Strategy

A key to the ongoing growth and development of the research unit is the organisations' ‘Research Strategy’ [12]. To the best of our searching, there are no published papers describing health service led research strategies for rural health services in areas classed as MMM 4–5. For research within rural health services to be accepted and encouraged as ‘standard business’, based on the experience at CAH, we suggest health services consider some key pillars of focus to address barriers to research in rural settings.

## 3 Suggested Key Pillars of a Rural Health Service ‘Research Strategy’

- Maintain and build on existing research partnerships (e.g., local UDRHs and Research Translation Centres who have significant coverage across rural and remote Australia [13]).
- Build a research profile within the health service, so non‐research staff are aware of opportunities.
- Enable staff to engage in research and enhance skills, promoting equity and diversity of opportunities across disciplines.
- Embed a strong research culture within departments, roles and teams.
- Ensure partnership and collaboration with consumers.
- Align activity with the Quality and Safety department to ensure cross‐collaboration, innovation and translation of research findings to relevant policy and practice.

**FIGURE 1:**
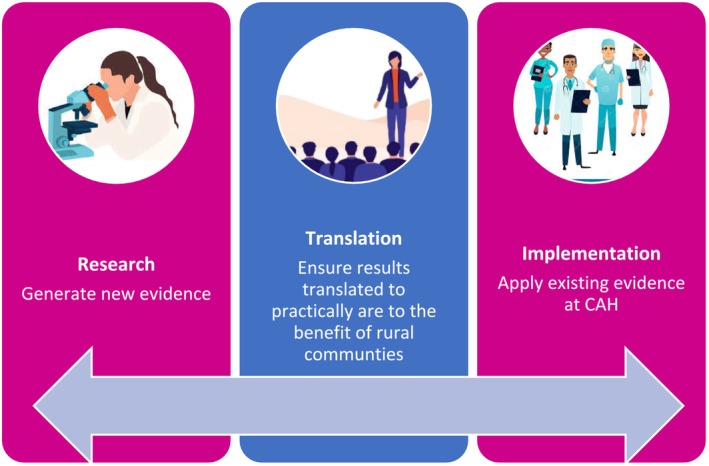
Key focus areas within the research strategy.

The ‘Research Strategy’ serves as an organisational policy document that outlines the organisations commitment to providing an environment that supports and utilises research and the overall goals of the strategy in practical terms (Figure 1). The research strategy also aligns with key federal programs, including RHMT, supporting rural health research (Parameter 5, maintaining and progressing an evidence base and the rural health agenda) and supporting environments whereby rural students can also benefit from access to research opportunities and resources [11]. The integration of research into rural health services and the opportunities it supports for training rural clinicians also aligns with Federal Government rural health workforce strategies [14]. Support for similar rural health service research unit models would constitute a practicable research enabler for any future state and federal rural health research strategies [3].

## 4 Alignment With Quality and Safety

The research strategy outlines a commitment to ensuring research activity aligns with the Quality and Safety priorities of the organisation. Through close collaboration between the Quality and Safety Department, Executive staff, the local UDRH, Western Alliance and the research unit, we have developed ‘Quality, Safety and Research’ project roles. These roles also act as a workforce retention strategy and are situated within the Quality department and focus on key areas of interest to Quality and Safety. They offer an opportunity for a local rural clinician to build skills in quality and safety and combine this with research skill development. The evaluation of these roles is currently underway. The research and quality departments also regularly hold ‘Innovation Hub’ meetings that are open to employees who would like to discuss key quality and safety issues, alongside generating research ideas from these.

## 5 Challenges and Areas for Further Research

There is a need to generate evidence on how models, such as this could be further systematised and integrated with other rural health services settings. There is also a risk of staff burn out given there are a small number of staff members with research skills, so realistic goals and workload management must be considered [15].

## 6 Conclusion

This *Practice Insight* demonstrates that rural health services can integrate research units into their organisational structures, with minimal burden on resources alongside support from partners who understand the value of rural health research. Commitment from health service leaders, funding support and collaboration with career researchers, along with defined organisational research strategy, have supported the ongoing growth of the research unit in this context. Lessons learned serve as a valuable example for other rural health services who are ambitiously seeking to drive their own research programs, build evidence on key issues and support their staff to access research career opportunities.

## Author Contributions

**Laura Alston:** conceptualization, investigation, writing – original draft, writing – review and editing, funding acquisition. **Michael Field:** conceptualization, investigation, funding acquisition, writing – review and editing. **Alison Buccheri:** conceptualization, investigation, funding acquisition, writing – review and editing. **Fiona Brew:** conceptualization, investigation, funding acquisition, writing – review and editing, supervision. **Anna George:** conceptualization, investigation, funding acquisition, writing – review and editing. **Nikita Wheaton:** conceptualization, investigation, writing – review and editing. **Stella Harrington:** conceptualization, investigation, writing – review and editing. **Warren Payne:** conceptualization, investigation, funding acquisition, writing – review and editing. **Drew Aras:** conceptualization, investigation, funding acquisition, writing – review and editing. **Alice Bennett:** conceptualization, investigation, writing – review and editing, funding acquisition. **Hannah Beks:** conceptualization, investigation, writing – review and editing. **Kevin Mc Namara:** conceptualization, investigation, funding acquisition, writing – review and editing. **Vincent L. Versace:** conceptualization, investigation, funding acquisition, writing – review and editing, supervision.

## Conflicts of Interest

The authors declare no conflicts of interest.

## Data Availability

The data that support the findings of this study are available from the corresponding author upon reasonable request.
